# Allostasis in health and food addiction

**DOI:** 10.1038/srep37126

**Published:** 2016-11-23

**Authors:** Dirk De Ridder, Patrick Manning, Sook Ling Leong, Samantha Ross, Sven Vanneste

**Affiliations:** 1 Section of Neurosurgery, Department of Surgical Sciences, Dunedin School of Medicine, University of Otago, New Zealand; 2 Section of Endocrinology, Department of Medicine, Dunedin School of Medicine, University of Otago, New Zealand; 3 School of Behavioral and Brain Sciences, University of Texas at Dallas, USA

## Abstract

Homeostasis is the basis of modern medicine and allostasis, a further elaboration of homeostasis, has been defined as stability through change, which was later modified to predictive reference resetting. It has been suggested that pleasure is related to salience (behavioral relevance), and withdrawal has been linked to allostasis in addictive types. The question arises how the clinical and neural signatures of pleasure, salience, allostasis and withdrawal relate, both in a non-addicted and addicted state. Resting state EEGs were performed in 66 people, involving a food-addicted obese group, a non-food addicted obese group and a lean control group. Correlation analyses were performed on behavioral data, and correlation, comparative and conjunction analyses were performed to extract electrophysiological relationships between pleasure, salience, allostasis and withdrawal. Pleasure/liking seems to be the phenomenological expression that enough salient stimuli are obtained, and withdrawal can be seen as a motivational incentive because due to allostatic reference resetting, more stimuli are required. In addition, in contrast to non-addiction, a pathological, non-adaptive salience attached to food results in withdrawal mediated through persistent allostatic reference resetting.

The concept of homeostasis is fundamental to our understanding of how normal physiological processes are regulated. It encapsulates the body’s ability to maintain all the parameters of the organism’s internal milieu within limits permitting an organism to survive^1^. It was proposed that survival depended on two important mechanisms: those required to maintain a physiological steady state (homeostasis) and those necessary to meet with sudden external demands (emergency)^2^. In other words, the internal environment (*milieu intérieur*) has to be maintained in balance with the external environment^2^.

Homeostasis is predominantly based on negative feedback mechanisms which are not particularly adaptive to an ever-changing environment, especially since multicellular creatures developed mobility. In these circumstances predictive sensory stimuli allow for reference resetting of homeostatic systems to better adjust to a continuously changing environment^3^. This mechanism has been termed allostasis, which can be thought of as “stability through change”^4^. Allostasis is important because it permits an adjustment of a reference or set point to predicted demands based on memory and context^3^. The predictive component of allostasis is the fundamental difference between it and homeostasis, which is only responsive. Proposed advantages of allostatic mechanisms include (1) errors are reduced in magnitude and frequency, (2) response capacities of different components are matched, (3) resources are shared between systems to minimize reserve capacities and (4) errors are remembered and used to reduce future errors^3^.

Initially allostasis was considered a pathological process^5^. For example, in addiction the degree of pleasure experienced by an addicted substance decreases for the same amount of substance over time, resulting in a progressively greater intake of the addicted substance for an ever decreasing hedonic response. In other words, the hedonic reference resetting was leading to addiction^5^. However, it has recently been suggested that allostasis is a normal physiological response to maintain stability when parameters are outside of the normal homeostatic range by resetting the system parameters to a new set point^4^,^5^,^6^.

The underlying neurobiological and neurophysiological substrate of allostasis has yet to be defined. At a systems level, the insula and anterior cingulate have been implicated in pain allostasis^7^,^8^.

Obesity can be thought of as a change in the reference or homeostatic set point for body weight or energy input. Although controversial, it has also been suggested that at least a sub-set of obese individuals may have an addictive tendency towards food^9^,^10^. Recently a questionnaire has been developed that is capable of identifying eating patterns that are similar to behaviors seen in classic areas of addiction^11^,^12^: substance taken in a larger amount and for a longer period than intended; persistent desire or repeated unsuccessful attempts to quit; significant time/activity elicited to obtain, use, or recover; important social, occupational, or recreational activities given up or reduced; use continues despite knowledge of adverse consequences; tolerance; characteristic withdrawal symptoms; substance taken to relieve withdrawal; and use that causes clinically significant impairment or distress.

It has been suggested that in food addiction ‘wanting’, which has been coined incentive salience^13^, becomes sensitized and dissociated from ‘liking’, which typically remains unchanged or may develop a blunted pleasure response to the food^14^. The result is excessive food intake despite minimal pleasure being related to withdrawal, which can be seen as a motivational incentive to take in more food^14^.

Food intake must have behavioral relevance (i.e. salience) in both lean and obese people, as energy intake is required to stay alive. In food addiction, it is hypothesized that food gains an abnormal or paradoxical salience, and it is considered behaviorally important even if enough food has been taken in to satisfy energy requirements. This paradoxical salience could reset the reference or set point for satiety when obtaining food which will subsequently drive more food intake. Furthermore, the reference resetting for satiety (allostasis) could also lead to withdrawal in the absence of the atypical behaviorally important food stimulus, further increasing food intake. This leads to the prediction that in food addiction salience and allostasis are related, in contrast to non-food addiction, which can be tested experimentally. In this study we clinically investigate how pleasure, salience, allostasis and withdrawal are related based on behavioral self-reports from obese people with food addiction, obese people without food addiction, and lean individuals. Furthermore, we look at brain activity and connectivity correlates of pleasure, salience, allostasis and withdrawal and analyze how they relate by looking at overlapping and differential activity and connectivity.

## Methods and Materials

### Research participants

Twenty healthy normal weight adults and 46 obese participants (see Table 1 for baseline characteristics) were recruited from the community by way of a newspaper advertisement. Inclusion criteria included male or female participants aged between 20 and 65 years and a BMI 19–25 kg/m^2^ (lean group) or >30 kg/m^2^ (obese group). Participants were excluded if they had other significant co-morbidities including diabetes, malignancy, cardiac disease, uncontrolled hypertension, psychiatric disease, previous head injury or any other significant medical condition.

The 20 healthy normal weight adults with BMIs between 18.5 and 24.9 were recruited to serve as a control group to verify what the neural correlates for pleasure, salience, allostasis and withdrawal are in a normal weight, non-food addicted group and how food addicted and non-food addicted obese people differ in their brain activity and functional connectivity with healthy non-obese controls.

### Procedures

All potential participants attended the research facilities for a screening visit and to undertake informed consent procedures. The study protocol had been approved and carried out in accordance by the Southern Health and Disability Ethics Committee (LRS/11/09/141/AM01). All participants underwent anthropometric measurements, physical examinations and resting energy expenditure and body composition analyses. Subsequently, those participants who met inclusion criteria reported to the facility after an overnight fast for EEG analysis, blood collection and questionnaire assessments.

### Questionnaire assessments

*YFAS.* Yale Food Addiction Scale (YFAS) is a self-reported standardized questionnaire, based on DSM-IV codes for substance dependence criteria, to identify individuals at high risk for food addiction, regardless of body weight^12^,^15^,^16^. While there is currently no official diagnosis of “food addiction”, the YFAS was created to identify persons who exhibited symptoms of dependency towards certain foods. The YFAS is a psychometrically validated tool consisting of 27 questions that identifies eating patterns that are similar to behaviors seen in classic areas of addiction^12^. The YFAS can also be divided into 8 subscales with domains similar to those of substance use disorder: substance taken in a larger amount and for a longer period than intended; persistent desire or repeated unsuccessful attempts to quit; significant time/activity elicited to obtain, use, or recover; important social, occupational, or recreational activities given up or reduced; use continues despite knowledge of adverse consequences; tolerance; characteristic withdrawal symptoms; substance taken to relieve withdrawal; and use that causes clinically significant impairment or distress. Using the continuous scoring system scale we calculated a YFAS score out of 7 for each participant (2). But in order to dichotomize the continuous scale into a food addicted vs non-food addicted group we performed a median split, with a low and high YFAS group, so that neural correlates of pleasure, salience, allostasis and withdrawal in food addicted obesity can be compared to non-food addicted obesity and a lean control group. Thus a median-split was applied on the YFAS for the obesity group. Eight participants had a score equal to the median (=3) and were excluded from the analysis. Participants with a score lower than the median were assigned to the low YFAS group, while those with a score higher than the median were assigned to the high YFAS group.

### Assessment of general addictive tendencies

The general addictive tendency of food addicted people over multiple domains was investigated using the general addictive tendencies questionnaire (GATQ). This is based on the concept of addiction transfer, i.e. when one addiction is treated, e.g. food addiction by gastric surgery, that addicted people sometimes become addicted to other substances or present with other addictive behavior^17^.

Based on the available literature that there may be a universal pathophysiological mechanism underlying addiction/substance abuse in general^18^, we are interested in finding the neural correlates of pleasure, salience, allostasis and withdrawal in general in the addicted brain, as well as in people without addictive tendencies. We therefore used a modified version of the general addictive tendencies questionnaire^19^. The questionnaire scores high on reliability and has good construct validity^19^. Four addiction-related items were recorded for each of the following 12 domains: alcohol, cigarettes, drugs, caffeine, chocolate, exercise, gambling, music, internet, shopping, work and love/relationships. These addiction-related items were (1) whether participants regarded the substance/activity as behaviorally important (salience), (2) whether they regarded it as enjoyable (pleasure), (3) whether they felt a need to consume more/engage in it more to achieve the same effect (allostasis) and (4) whether they feel discomfort when they discontinue use (withdrawal). Five-point response scales ranging from (1) very false for me to (5) very true for me were used for each item. All addiction-related scales have high levels of internal consistency reliability (e.g., for the total 96-item addiction scale, alpha = 0.93). The average scores for each of the 4 addiction related items (pleasure, salience, allostasis and withdrawal) were calculated across all 12 domains, as to represent a true score for a general addictive tendency.

### Statistics

A comparison between the lean, the low YFAS and high YFAS group was conducted using an ANOVA using group association as the independent variable and the 8 domains of the YFAS as dependent variables. In addition, we applied a Pearson correlation between the four measures of general addictive tendencies for the whole group, as well as for the lean, the low YFAS and high YFAS groups separately. In addition, we performed a mediation regression analysis^20^ on the high YFAS group to have a better understanding of the relationship between salience, allostasis and withdrawal. Rather than a direct causal relationship between the independent variable (salience) and the dependent variable (withdrawal), a mediation model was computed to determine whether the independent variable (salience) influences the mediator variable (allostasis), which in turn influences the dependent variable (withdrawal).

### Imaging data

#### EEG Data collection

Resting state EEGs were recorded, as the authors were interested in elucidating the neural correlates of pleasure, salience, allostasis and withdrawal as underlying mechanisms present in the (food) addicted brain. The hypothesis is that there are neural signatures in the brain, even when (food) addicted people are not exposed to the substance of abuse (food), that can be detected, which predispose people to (food) addiction.

EEG data were recorded per standard procedure. Recordings were performed in a fully lighted room with each participant sitting upright on a small but comfortable chair. The actual recording lasted approximately five minutes. Patients were instructed to sit still and relax their jaws and neck with their eyes closed, focusing on one point in front of them. The EEG was sampled using Mitsar-201 amplifiers (NovaTech http://www.novatecheeg.com/) with 19 electrodes placed according to the standard 10–20 International placement (Fp1, Fp2, F7, F3, Fz, F4, F8, T7, C3, Cz, C4, T8, P7, P3, Pz, P4, P8, O1, O2). Participants abstained from alcohol consumption 24 hours prior to EEG recording and from caffeinated beverages on the day of recording in order to avoid alcohol-induced changes in EEG^21^ or a caffeine-induced alpha power decrease^22^,^23^. The vigilance of participants was monitored by EEG parameters such as the slowing of alpha rhythm or the appearance of spindles as drowsiness is reflected in enhanced theta power^24^. Impedances were checked to remain below 5 kΩ. Data were collected with eyes-closed (sampling rate = 500 Hz, band passed 0.15–200 Hz). Off-line data were resampled to 128 Hz, band-pass filtered in the range 2–44 Hz and subsequently transposed into Eureka! software^25^, plotted and carefully inspected for manual artifact-rejection. All episodic artifacts including eye blinks, eye movements, teeth clenching, body movement, or ECG artifact were removed from the stream of the EEG. In addition, an independent component analysis (ICA) was conducted to further verify if all artifacts had been excluded. To investigate the effect of possible ICA component rejection, we compared the power spectra with two approaches: (1) after visual artifact rejection only, and (2) after additional ICA component rejection. The mean power in delta (2–3.5 Hz), theta (4–7.5 Hz), alpha1 (8–10 Hz), alpha2 (10–12 Hz), beta1 (13–18 Hz), beta2 (18.5–21 Hz), beta3 (21.5–30 Hz) and gamma (30.5–44 Hz) bands^26^,^27^,^28^ did not show a statistically significant difference between the two approaches. We were therefore confident in reporting the results of two-step artifact correction data, namely visual artifact rejection and additional independent component rejection. Average Fourier cross-spectral matrices were computed for all eight bands.

#### Source localization

Standardized low-resolution brain electromagnetic tomography (sLORETA^29^,^30^) was used to estimate the intracerebral electrical sources that generated the seven group BSS components. As standard procedure, a common average reference transformation^29^ was performed before applying the sLORETA algorithm. sLORETA computes electric neuronal activity as current density (A/m2) without assuming a predefined number of active sources. The solution space used in this study and associated leadfield matrix are those implemented in the LORETA-Key software (freely available at http://www.uzh.ch/keyinst/loreta.htm). This software implements revisited realistic electrode coordinates and the lead field produced by applying the boundary element method on the MNI-152 (Montreal neurological institute, Canada) template of Mazziotta *et al.*^31^,^32^. The sLORETA-key anatomical template divides and labels the neocortical (including hippocampus and anterior cingulate cortex) MNI-152 volume in 6,239 voxels of dimension 5 mm^3^, based on probabilities returned by the Demon Atlas^33^,^34^. The co-registration makes use of the correct translation from the MNI-152 space into the Talaiach and Tournoux^35^ space^36^.

#### Whole brain correlation analysis

Correlations are calculated for pleasure, withdrawal, allostasis and salience with brain activity. The methodology used for the sLORETA correlations is non-parametric. It is based on estimating, via randomization, the empirical probability distribution for the max-statistic, under the null hypothesis comparisons^37^. This methodology corrects for multiple testing (i.e., for the collection of tests performed for all voxels, and for all frequency bands). Due to the non-parametric nature of the method, its validity does not rely on any assumption of Gaussianity^37^. sLORETA statistical contrast maps were calculated through multiple voxel-by-voxel comparisons. The significance threshold was based on a permutation test with 5000 permutations.

#### Conjunction analysis

We conducted a conjunction analysis with the whole brain correlation measures of pleasure, withdrawal, allostasis and salience^38^,^39^,^40^,^41^. A conjunction analysis identifies a ‘common processing component’ for two or more tasks/situations by finding areas activated in independent subtractions^38^,^39^,^40^,^41^. Friston *et al.*^39^ also indicated that although general conjunction analysis is used in a within group condition, it can also be applied between groups and was applied in some recent papers^42^,^43^.

#### Whole brain comparison analysis

In order to identify potential differences in brain electrical activity between low and high YFAS obese participants, sLORETA was then used to perform voxel-by-voxel between-condition comparisons of the current density distribution. Nonparametric statistical analyses of functional sLORETA images were performed for each contrast employing an F-statistic for unpaired groups and corrected for multiple comparisons. As explained by Nichols and Holmes, the SnPM methodology does not require any assumption of Gaussianity and corrects for all multiple comparisons^37^. We performed one voxel-by-voxel test (comprising 6,239 voxels each) for the different frequency bands.

#### Lagged Phase Coherence

Coherence and phase synchronization between time series corresponding to different spatial locations are usually interpreted as indicators of the “connectivity”. However, any measure of dependence is highly contaminated with an instantaneous, non-physiological contribution due to volume conduction^44^. However, Pascual-Marqui^45^, introduced new measures of coherence and phase synchronization taking into account only non-instantaneous (lagged) connectivity, effectively removing the confounding factor of volume conduction. Such “lagged phase coherence” between two sources can be interpreted as the amount of cross-talk between the regions contributing to the source activity^46^. Since the two components oscillate coherently with a phase lag, the cross-talk can be interpreted as information sharing by axonal transmission. More precisely, the discrete Fourier transform decomposes the signal in a finite series of cosine and sine waves at the Fourier frequencies (Bloomfield 2000). The lag of the cosine waves with respect to their sine counterparts is inversely proportional to their frequency and amounts to a quarter of the period; for example, the period of a sinusoidal wave at 10 Hz is 100 ms. The sine is shifted a quarter of a cycle (25 ms) with the respect to the cosine. Then the lagged phase coherence at 10 Hz indicates coherent oscillations with a 25 ms delay, while at 20 Hz the delay is 12.5 ms, etc. The threshold of significance for a given lagged phase coherence value according to asymptotic results can be found as described by Pascual-Marqui (2007), where the definition of lagged phase coherence can be found as well. As such, this measure of dependence can be applied to any number of brain areas jointly, i.e., distributed cortical networks, whose activity can be estimated with sLORETA. Measures of linear dependence (coherence) between the multivariate time series are defined. The measures are non-negative, and take the value zero only when there is independence and are defined in the frequency domain: delta (2–3.5 Hz), theta (4–7.5 Hz), alpha1 (8–10 Hz), alpha2 (10–12 Hz), beta1 (13–18 Hz), beta2 (18.5–21 Hz), beta3 (21.5–30 Hz) and gamma (30.5–44 Hz). Based on this, principle lagged linear connectivity was calculated. Time-series of current density were extracted for different region of interests using sLORETA. Power in all 6,239 voxels was normalized to a power of 1 and log transformed at each time point. The results are reported using a F-test and reported as the log of the F-ratio. Region of interest values thus reflect the log transformed fraction of total power across all voxels, separately for specific frequencies. Regions of interest selected were the pregenual anterior cingulate cortex, the dorsal anterior cingulate cortex and the posterior cingulate cortex.

#### Statistical analyses for the lagged phase coherence

Lagged phase synchronization/coherence for functional connectivity contrast maps were calculated. Comparison was computed between the addicted and control groups as well as correlated with allostasis, withdrawal and salience for the high YFAS group. The significance threshold was based on a permutation test with 5000 permutations. This methodology corrects for multiple testing (i.e. for the collection of tests performed for all voxels, and for all frequency bands). The results are reported using a F-test and reported as the log of the F-ratio.

## Results

### Participant characteristics

**Figure 1:**
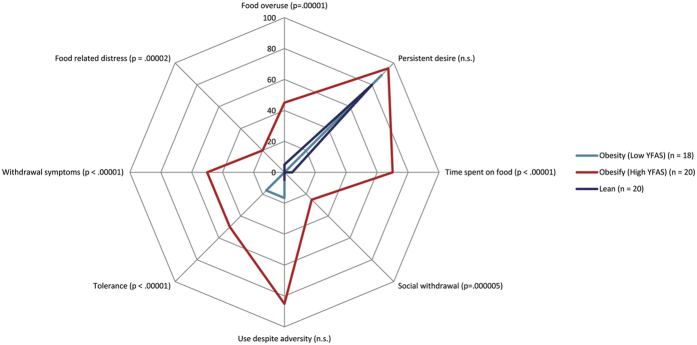
Radar image representing percentage of people exhibiting each food related symptom. The food addicted obese group (high YFAS) behaves differently from the lean and the non-food addicted obese group (low YFAS). The lean and non-food addicted group show exactly the same food related behaviour.

In general, a comparison between the lean, low and high YFAS shows a significant difference (*F* = 104.18, *p* < 0.001). The lean group and the low YFAS do not differ from each other, but do differ from the high YFAS group. This was confirmed by different subscales of the YFAS: food overuse, time spent on food, social withdrawal, withdrawal symptoms and food (see Fig. 1); however, the high YFAS group does not differ from the low YFAS or lean groups regarding persistent use despite adversity or tolerance.

### Behavioral Data

**Figure 2:**
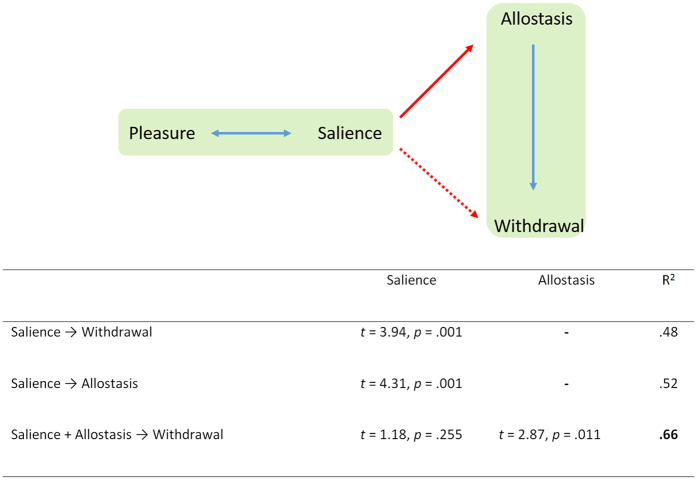
Pleasure is related to salience in all groups, as is allostasis to withdrawal. However, salience is related to allostasis and withdrawal only in the addicted group. Furthermore, the influence of salience on withdrawal is indirect, mediated via allostasis.

A correlation analysis between the four subscales of the general addictive tendencies questionnaire revealed a significant positive correlation (after correction) between pleasure and salience as well as between allostasis and withdrawal for all three participant groups (see Table 2). A similar relationship was identified between pleasure and salience as well as between allostasis and withdrawal for lean and low YFAS participants separately. For the high YFAS group a significant positive correlation was found between both pleasure and salience and between allostasis and withdrawal. A positive correlation was also identified between salience and allostasis as well as between salience and withdrawal for the same group. A mediation effect further showed that the relationship between salience and withdrawal was mediated by allostasis (*Sobel test:* 3.17, *p* = 0.001; see Fig. 2).

### Imaging data

#### Whole brain correlation analysis: pleasure, withdrawal, allostasis and salience (whole group: lean, low and high YFAS)

**Figure 3:**
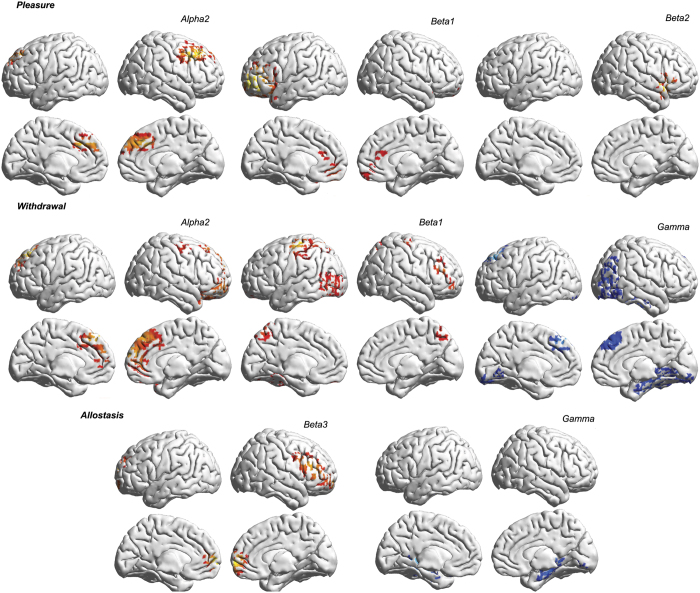
Correlation analyses between pleasure (top panel), withdrawal (mid panel), allostasis (bottom panel) and source localized (sLORETA) brain activity. Warm colors (yellow-red) represent positive correlations, cold colors (blue) represent negative correlations.

A correlation analysis between pleasure and brain activity revealed a significant positive correlation between alpha2 activity in the rostral anterior cingulate cortex extending into the dorsomedial prefrontal cortex and dorsolateral prefrontal cortex (Fig. 3). A positive correlation was also identified between pleasure and beta1 frequency band activity in the pregenual anterior cingulate cortex and ventrolateral prefrontal cortex and beta2 frequency activity in the right insula (Fig. 3). No significant effect was identified for the delta, theta, alpha1, beta3 or gamma frequency bands.

A significant positive correlation was identified between withdrawal and alpha2 frequency band activity in the rostral anterior cingulate cortex/dorsal medial prefrontal cortex (Fig. 3). A positive correlation was seen between withdrawal and beta1 frequency band activity in the precuneus, the dorsolateral prefrontal cortex, superior parietal lobe and the left temporo-occipital junction. A negative correlation was identified between withdrawal and gamma band activity in the dorsomedial prefrontal cortex and parahippocampal area, and the right temporoparietal area. No significant effect was identified for the delta, theta, alpha1, beta2 or beta3 frequency bands.

Allostasis correlated positively with beta3 activity in the pregenual anterior cingulate cortex and dorsolateral prefrontal cortex and negatively with gamma band activity in the left parahippocampus (Fig. 3). No significant effect was identified for the delta, theta, alpha1, alpha2, beta1 or beta2 frequency bands.

No significant correlations were identified between salience and activity in any of the frequency bands.

#### Conjunction Analysis (whole group)

**Figure 4:**
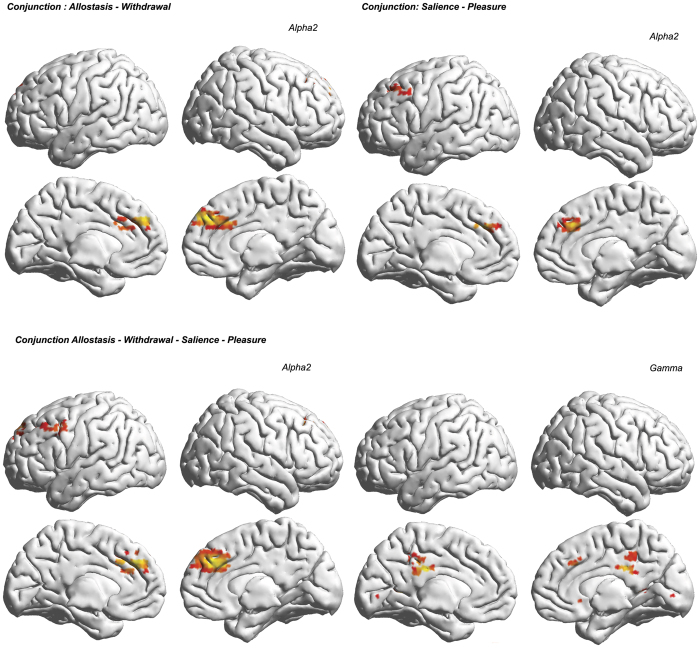
Conjunction analyses for food-addicted, non-food-addicted and lean individuals between allostasis and withdrawal (top panel, left), between pleasure and salience (top panel, right) and between allostasis, withdrawal, pleasure and salience (lower panel).

A conjunction analysis between allostasis and withdrawal showed shared bilateral alpha2 activity in the rostral anterior cingulate cortex/dorsal medial prefrontal cortex. No effect was identified for the delta, theta, alpha1, beta1, beta2, beta3 or gamma frequency bands (Fig. 4, top left panel).

A conjunction analysis between salience and pleasure also showed common alpha2 activity in the rostral anterior cingulate cortex/dorsal medial prefrontal cortex (Fig. 4, top right panel). No effect was identified for the delta, theta, alpha1, beta1, beta2, beta3 or gamma frequency bands.

A conjunction analysis of the two abovementioned conjunction analyses showed common bilateral alpha2 activity in the rostral anterior cingulate cortex/dorsal medial prefrontal cortex and common gamma band activity in the left rostral anterior cingulate cortex/dorsal medial prefrontal cortex, the dorsal lateral prefrontal cortex and bilateral posterior cingulate cortex (Fig. 4, lower panel). No effect was identified for the delta, theta, alpha1, beta1, beta2, or beta3 frequency bands.

#### Low vs High YFAS comparison

**Figure 5:**
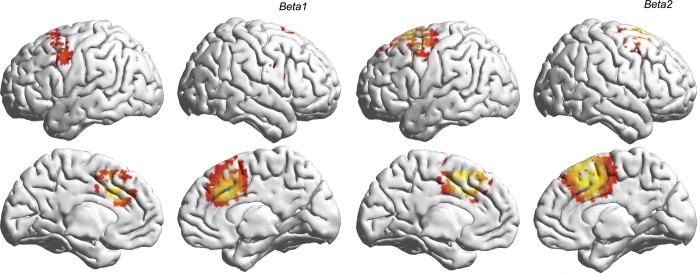
A comparison between low (non-addicted to food) and high YFAS (food addicted) participants shows increased beta1 and beta2 activity in the rACC/dmPFC bilaterally as well as in the premotor/motor cortex on the left for the high YFAS group.

A comparison between low (non-addicted to food) and high YFAS (food addicted) participants shows increased beta1 and beta2 activity in the rostral anterior cingulate cortex/dorsal medial prefrontal cortex bilaterally as well as in the premotor/motor cortex on the left for the high YFAS group (Fig. 5). No effect was identified for the delta, theta, alpha1, alpha2, beta3, or gamma frequency bands.

#### Conjunction analysis (High YFAS group)

**Figure 6:**
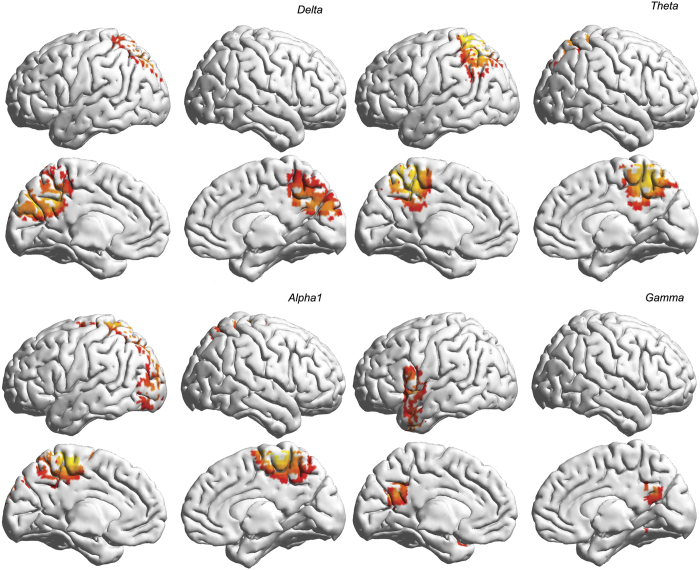
A conjunction analysis for the High YFAS participants between salience and allostasis demonstrates shared activity in the posterior cingulate cortex extending to the precuneus for the delta, theta, and alpha1 band. In addition, for the theta frequency band shared activity was identified in the superior parietal lobe. For the gamma band shared activity is noted in the PCC bilaterally as well as in the left VLPFC, insula and anterior temporal pole (lower right quadrant of Fig. 5).

A conjunction analysis for the High YFAS participants between salience and allostasis demonstrated shared activity in the posterior cingulate cortex extending to the precuneus for the delta, theta, and alpha1 bands (Fig. 6). In addition, for the theta frequency band, shared activity was identified in the superior parietal lobe. For the gamma band, shared activity was noted in the posterior cingulate cortex bilaterally as well as in the left ventral lateral prefrontal cortex, insula and anterior temporal pole (lower right quadrant of Fig. 6). No effect was identified for the delta, alpha2, beta1, or beta2 frequency bands.

#### Group comparisons for lagged phase coherence

**Figure 7:**
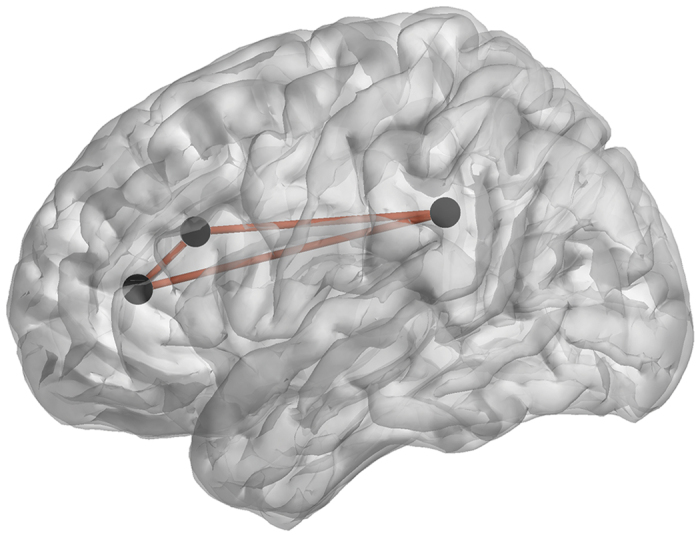
For the gamma frequency band, a comparison between the addicted group and the control group shows a significant increased connectivity (the log of *F*-ratio = 1.76, *p* < 0.05) between the pregenual anterior cingulate cortex, dorsal anterior cingulate cortex and the posterior cingulate cortex for the addicted group.

Significantly increased connectivity (*F* = 1.76, *p* < 0.05) was identified between the pregenual anterior cingulate cortex, dorsal anterior cingulate cortex and the posterior cingulate cortex for the gamma frequency band for the High YFAS group compared to the control group (see Fig. 7). No significant effect was identified for delta, theta, alpha1, alpha2, beta1, beta2 or beta3 frequency bands.

#### Lagged phase coherence correlation analysis for the high YFAS group

**Figure 8:**
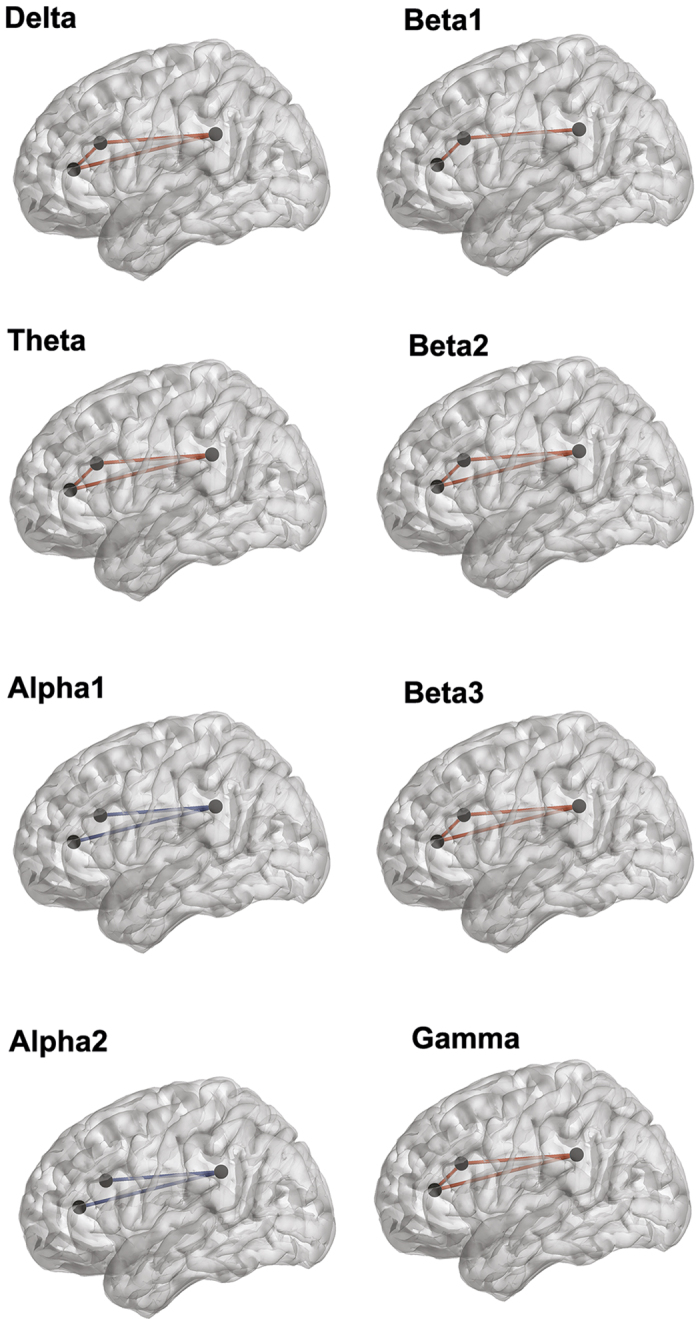
A correlation analysis between the lagged phase coherence and allostasis showed a significant effect (r = 0.38, *p* < 0.05) for delta, theta, alpha1, alpha2, beta1, beta2, beta3 and gamma frequency band for the addicted group.

A correlation analysis between the lagged phase coherence and allostasis showed a significant effect (r = 0.38, *p* < 0.05) for the delta, theta, alpha1, alpha2, beta1, beta2, beta3 and gamma frequency bands. For the delta, theta, beta2, beta3 and gamma frequency bands an increased connection was identified between the pregenual anterior cingulate cortex, dorsal anterior cingulate cortex and the posterior cingulate cortex. This suggests that the higher the addicted participants score on allostasis, the stronger the connectivity is between the three areas. For the alpha1 and alpha2 frequency bands, a decreased connectivity was identified between pregenual anterior cingulate cortex and the posterior cingulate cortex as well as between the dorsal anterior cingulate cortex and the posterior cingulate cortex. This indicates that the lower the addicted participants score on allostasis, the stronger the connectivity is. For the beta1 frequency band a significant effect was identified between the dorsal anterior cingulate cortex and the posterior cingulate cortex as well as between pregenual anterior cingulate cortex and dorsal anterior cingulate cortex. This latter finding suggests that the higher addicted participants score on allostasis, the stronger the associated connectivity is. See Fig. 8 for an overview.

A correlation analysis between the lagged phase coherence and respectively withdrawal and salience revealed no significant effects for the delta, theta, alpha1, alpha2, beta1, beta2, beta3 or gamma frequency bands.

## Discussion

Our self-reported behavioral results suggest that the pleasure derived from a substance or activity is related to the salience, or behavioral relevance, attributed to it. In addition, it appears that predictive reference resetting (allostasis) is strongly related to withdrawal. These associations are present for both food addicted and non-food addicted individuals, indicating that they are a normal physiological response. Indeed, when taking in food, exactly the same food stimulus at the beginning of the meal (when hungry) has a different hedonic weight attached to it than at the point in the meal when satiety has set in. This suggests that allostasis, i.e. reference resetting, occurs physiologically, so that people stop eating once the bodily energy requirements are fulfilled. In other words, allostasis is state or context dependent. In non-food addicted individuals or lean people salience does not influence allostasis, but it does in those with food addiction, suggesting that this is a pathological phenomenon which could be a characteristic of food addiction. This suggests that in people with food addiction, behavioral relevance (i.e. salience) of the substance (of abuse) drives a predictive reference resetting (i.e. allostasis) that results in a desire to obtain more of the substance (craving) which runs parallel to the negative motivational state known as withdrawal^47^.

Interestingly, the neuroimaging results suggests that pleasure, salience, allostasis and withdrawal are all related neurophysiologically, as they share a common hub in the rostral anterior cingulate cortex/dorsal medial prefrontal cortex and dorsolateral prefrontal cortex, as well as in the posterior cingulate cortex as demonstrated by the conjunction analyses. This is common to both the food addicted, non-food addicted and lean individuals, suggesting that it represents a normal physiological phenomenon.

The rostral anterior cingulate cortex is involved in “uncertainty” processing^48^,^49^,^50^,^51^,^52^. Uncertainty is defined as a state in which a given representation of the world cannot be adopted to guide subsequent belief^53^ and can be reduced by acquiring more information from the environment^51^ or by drawing on memory^54^. The rostral to dorsal anterior cingulate cortex has a role in acquiring new data in an attempt to reduce uncertainty^55^,^56^. It is therefore unsurprising that our results indicate that activity in the anterior cingulate region correlates with withdrawal, which will trigger an urge for action, encoded by the dorsal anterior cingulate cortex and insula^57^. The pregenual anterior cingulate cortex seems to suppress further input in the somatosensory^58^,^59^, vestibular^60^ and auditory systems^61^. Malfunctioning of this mechanism leads to a hyperactive state within these systems resulting in fibromyalgia related pain^62^, vertigo^60^ or tinnitus respectively^63^,^64^,^65^,^66^. Furthermore, the same area suppresses aggression^67^,^68^,^69^, and a genetically determined deficiency of pregenual anterior cingulate cortex control over the amygdala is related to aggressiveness^67^,^68^,^69^. Thus, the pregenual anterior cingulate cortex seems have a non-specific suppression function analogous to the non-specificity of the dorsal anterior cingulate cortex as part of a general salience network^70^,^71^ that functions to acquire more input^57^ by attaching salience to stimuli^70^,^72^,^73^. The pregenual anterior cingulate cortex also has an important role in encoding pleasure via its connection to the orbitofrontal cortex^74^. This is in keeping with the concept that pleasure is a common currency to prioritize processing of behaviorally relevant stimuli^75^,^76^. In this study, the amount of pleasure derived from the substance or action correlates to increasing activity in the pregenual anterior cingulate and rostral anterior cingulate cortices extending into the dorsal lateral prefrontal cortex (see Fig. 3).

Our results point to allostasis being a normal physiological process, confirming the findings of others^3^. This predictive reference resetting mechanism appears to be controlled by the rostral anterior cingulate cortex and the dorsal lateral prefrontal cortex as demonstrated by the neuroimaging data of this study. Importantly, our data suggest that allostasis also drives physiological withdrawal as it is a common finding in lean as well as all obese individuals. It would thus appear that withdrawal induced wanting relates to allostasis in a similar fashion as “liking”/pleasure relates to salience.

In lean and non-food addicted individuals, salience and withdrawal are unrelated. In contrast, in food addicted individuals, salience modifies withdrawal; however, this effect appears to be mediated indirectly, via allostatic reference resetting. Thus, food addiction seems to be characterized by a selective interaction between salience and allostasis. The question then becomes: what neural mechanism underlies this pathological salience-driven reference resetting? The conjunction analysis between salience and allostasis in the food addicted group indicates that this phenomenon is related to activity in the posterior cingulate cortex extending into the precuneus and the superior parietal lobule, as well as the ventral lateral prefrontal cortex extending into the insula and anterior temporal lobe. One could speculate that in the addicted state, posterior cingulate cortex involvement allows for resetting of the self-referential set point based on the salience of the stimulus. This is suggested by the functional connectivity between the PCC and the ACC (Fig. 6), which correlates with the amount of reference resetting (allostasis) (Fig. 7). The posterior cingulate cortex is the main hub of the self-referential default mode network^77^,^78^ and seems to be involved in allostasis (see Fig. 5). One of its core functions is to allow for adaptive changes of behavior in the face of a changing world^79^. Adapting to a changing environment requires that internal and external stimuli are predicted and then compared to the current state of the self. This likely occurs at different areas within the posterior cingulate cortex^80^,^81^. Indeed, processing of stimuli from the internal world predominantly occurs in the ventral posterior cingulate cortex, whereas processing of stimuli from the external world predominantly occurs at the dorsal posterior cingulate cortex^81^. Thus predictive reference resetting might critically depend on posterior cingulate activity and functional connectivity.

The critical behavioral difference between addiction and non-addiction is salience driven allostasis (red arrow Fig. 1), which is related to activity in the pregenual anterior cingulate cortex/ventral medial prefrontal cortex and inversely related to activity in the parahippocampal area. In other words, this indicates an increase in pleasure related to a substance and a concomitant decrease of its contextual influence^82^,^83^, as the parahippocampal area is predominantly involved in contextual processing^82^,^83^. This suggests that the substance of abuse becomes independent from its context. This could hypothetically explain why addicted people do not stop consuming the substance of abuse, as contextual influences become less influential in suppressing further input. This is specific for the addictive type, as a conjunction between salience and allostasis in non-addictive obese and lean people does not show any significant overlapping activity. This suggests that in the addictive type an abnormal salience, detached from its contextual relevance, drives predictive reference resetting, as to obtain more input to reduce uncertainty (did I take in enough food to fulfill my energy demands?), and that this is phenomenologically expressed as withdrawal, a negative emotional state which will drive craving, an intense desire to consume the substance. Even though in non-addicted people allostasis also drives withdrawal, it is in the addicted people only that the allostasis is dependent on the salience of the stimulus, and this reference resetting seems to be controlled by the posterior cingulate cortex.

An important question is whether the salience-driven allostasis, unique in addiction, is the result of an abnormal functional connectivity which develops in addiction between the hub of the salience network (rostral to dorsal anterior cingulate cortex) and the hub of the self-referential (allostasis) network (posterior cingulate cortex) (see Fig. 5).

However, allostasis itself seems to be correlated to pregenual anterior cingulate cortex/ventral medial prefrontal cortex activity, which is also part of the self-referential default mode network. Another conceptual way of looking at this is that the self-referential posterior cingulate cortex communicates with the dorsal anterior cingulate cortex, involved in acquiring more input, and the pregenual anterior cingulate cortex, involved in suppressing more, and that the reference resetting in the posterior cingulate cortex controls the balance between input gathering and input suppression^55^. Therefore, the functional connectivity between these 3 areas was analyzed. This demonstrated that food addicted obese individuals had increased functional connectivity between the rostral anterior cingulate cortex – pregenual anterior cingulate cortex – posterior cingulate cortex network when compared to controls. As both the pregenual anterior cingulate cortex and posterior cingulate cortex belong to the self-referential default mode network, the salience network seems to become intrinsically linked to the default mode, and the stronger the connectivity, the more reference resetting occurs (except for alpha). The findings of this study suggest that the salience or behavioral relevance attached to food in food addicted people might reset their reference set point in the pregenual anterior cingulate cortex mediated via the self-referential posterior cingulate cortex. As no effective connectivity measures were calculated, this can only be hypothesized from a mechanistic point of view derived from the mediation analysis.

A weakness of this study is that the concepts of pleasure, salience, allostasis and withdrawal are based on single questions rather than questionnaires; however, the questions seem to capture the essence of the concepts. (1) salience is defined by a question that specifically asks whether participants regarded the substance/activity as behaviorally important^71^,^84^, (2) pleasure is described by a question that specifically asks whether they regarded it as enjoyable, (3) allostasis is defined by a question that specifically asks whether they felt a need to consume more/engage in it more to achieve the same effect^3^,^5^ and (4) withdrawal is defined by a question that asks whether they feel discomfort when they discontinue consuming. Because these questions all seem to capture the definition of the studied concepts, we believe this approach to be valid, albeit without nuancing the studied concepts. An advantage of this approach is that by limiting the question to the definition of the concept, it separates the studied concepts better than in larger questionnaires where more overlapping questions might be asked. Further studies should evaluate whether the single questions which are used in this study are indeed reflecting the described behavior (pleasure, salience, allostasis and withdrawal). This could be done by adding more comprehensive questionnaires and performing correlation analyses between the single questions and the more comprehensive questionnaires.

Another weakness of the study is that due to the fact that most participants meet 3 or more criteria of the YFAS, most patients can be considered food addicted. Yet, to verify whether the more severely addicted participants were behaviorally and neurophysiologically different from less addicted and lean controls, a median split analysis was performed. Future studies should include larger sample sizes as well as more distinctive groups. In addition, we applied a median split for the YFAS, which could be considered a weakness. However, the median-split clear shows a differentiation on the YFAS. As Fig. 1 indicates low YFAS subjects have a similar profile to the lean subjects, whereas people who score high on the YFAS clearly have a different profile.

Another limitation of this study is the low resolution of the source localization inherently resulting from a limited number of sensors (19 electrodes) and a lack of subject-specific anatomical forward models. This is sufficient for source reconstruction but results in greater uncertainty in source localization and decreased anatomical precision, and thus the spatial precision of the present study is considerably lower than that of functional MRI. Nevertheless, sLORETA has received considerable validation from studies combining LORETA with other more established localization methods, such as functional Magnetic Resonance Imaging (fMRI)^85^,^86^, structural MRI^87^ and Positron Emission Tomography (PET)^88^,^89^,^90^ and was used in previous studies to detect specific activity e.g. activity in the auditory cortex^91^,^92^,^93^. Further sLORETA validation has been based on accepting as ground truth the localization findings obtained from invasive, implanted depth electrodes, which is demonstrated in several studies on epilepsy^94^,^95^ and cognitive ERPs^96^. It is worth emphasizing that deep structures such as the anterior cingulate cortex^97^, and mesial temporal lobes^98^ can be correctly localized with these methods. However, further research could improve spatial precision, and accuracy could be achieved using high-density EEG (e.g., 128 or 256 electrodes), subject-specific head models, and MEG recordings.

In summary, input gathering or suppression of input is based on a prediction of what is energetically required, with information gleaned from areas involved in acquiring more input (rostral to dorsal anterior cingulate cortex) and an area that suppresses further input (pregenual anterior cingulate cortex). The self-referential prediction based on the energy requirement determines the allostatic reference, which is controlled by the self-referential posterior cingulate cortex. Withdrawal is a signal that more input is required, and pleasure indicates that enough input has been identified. These feelings are adjusted based on the allostatic level, which in addicted people is determined by a non-adaptive (non-dynamic or fixed) salience attached to the substance. Thus pleasure/liking seems to be the phenomenological expression that enough salient stimuli are obtained, and withdrawal leading to wanting is due to allostatic reference resetting so that more stimuli are required. In addition, in contrast to non-addiction, a pathological non-adaptive salience attached to the substance of abuse results in withdrawal, which will create an urge for action to obtain more of the same stimulus. Further studies will need to confirm some of the proposed mechanisms described in this report. This can be done by looking at a dynamic model in which food or drink is given till satiety is reached and performing sequential EEGs at different moments in time correlated to the satiety state.

## Additional Information

**How to cite this article**: De Ridder, D. *et al.* Allostasis in health and food addiction. *Sci. Rep.* **6**, 37126; doi: 10.1038/srep37126 (2016).

**Publisher’s note:** Springer Nature remains neutral with regard to jurisdictional claims in published maps and institutional affiliations.

## Figures and Tables

**Table 1:** Demographics, anthropometric, laboratory measures and general addictive tendencies questionnaire for the lean and obese groups (mean, standard deviation and range).

	Lean	Obese	
	(n = 20)	Low YFAS (n = 18)	High YFAS (n = 20)
Age	37.95 (11.51)	50.56 (9.12)	47.40 (14.37)
Gender	♂ 8 ♀ 12	♂ 2 ♀ 16	♂ 2 ♀ 18
Body weight (kg)	67.60 (9.30)	103.81 (18.91)	106.69 (21.38)
Height (cm)	172.01 (10.38)	166.08 (6.90)	163.91 (6.26)
BMI	22.76 (2.11)	37.67 (6.64)	39.56 (6.50)
Systolic BP	116.85 (10.78)	129.41 (10.34)	134.35 (17.44)
Diastolic BP	75.00 (6.29)	82.88 (7.87)	83.00 (6.37)
Heart rate	65.05 (12.13)	71.47 (9.61)	73.85 (9.26)
Waist	78.75 (5.81)	112.56 (15.17)	118.55 (15.96)
Resting energy expenditure	1625.40 (289.67)	1686.53 (332.69)	1768.40 (234.10)
% body fat	22.93 (8.33)	44.79 (8.45)	46.57 (5.69)
Fat mass	15.11 (5.21)	47.29 (15.94)	50.04 (13.37)
Fat free mass	52.18 (11.16)	56.24 (8.16)	56.48 (11.31)
% trunk fat	21.56 (7.37)	42.84 (7.35)	44.43 (4.81)
Trunk fat mass	7.87 (2.76)	23.60 (6.94)	24.79 (5.75)
Trunk fat free mass	29.07 (5.72)	30.68 (4.08)	30.62 (5.27)
Cholesterol (mmol/L)	4.66 (1.01)	5.56 (1.01)	5.59 (0.92)
Triglycerides (mmol/L)	0.94 (0.40)	1.29 (0.53)	1.37 (0.64)
HDL (mmol/L)	1.57 (0.34)	1.39 (0.28)	1.40 (0.27)
LDL (mmol/L	2.66 (0.83)	3.58 (0.92)	3.58 (0.75)
GGT (U/L)	15.35 (9.78)	34.11 (28.55)	25.95 (13.92)
ALT (U/L)	17.85 (7.67)	27.00 (17.41)	24.30 (11.06)
AST (U/L)	21.45 (5.30)	26.06 (24.97)	22.25 (7.04)
Glucose (mmol/L)	—	5.06 (0.42)	5.17 (0.50)
GATQ			
Allostasis	22.75 (6.44; 13–36)	23.96 (6.29; 13–36)	22.31 (2.77; 12–30)
Salience	36.95 (3.81; 27–44)	35.85 (4.17; 29–44)	33.84 (4.17; 26–41)
Pleasure	41.35 (4.28; 34–52)	38.89 (3.75; 31–45)	38.00 (3.79; 31-45)
Withdrawal	25.60 (5.85; 17–41)	35.85 (3.28; 29-44)	33.84 (4.17; 26–41)

**Table 2:** Correlations between salience, pleasure, withdrawal and pleasure for the whole group, the lean group, the non-addicted and addicted group.

	Salience	Pleasure	Withdrawal
*Whole group*			
Allostasis	0.36	0.26	0.71*
Withdrawal	0.39	0.28	
Pleasure	0.56*		
*Lean*			
Allostasis	0.28	0.39	0.65*
Withdrawal	0.47	0.36	
Pleasure	0.56*		
*Non-addicted group (low YFAS)*			
Allostasis	0.24	0.21	68.0*
Withdrawal	0.24	0.26	
Pleasure	0.53*		
*Addicted group (high YFAS)*			
Allostasis	0.72*	0.38	0.79*
Withdrawal	0.69*	0.24	
Pleasure	0.56*		

^*^Significant after Bonferroni correction (p < 0.05).
